# Coverage of Axillary Lymph Nodes with Tangential Breast Irradiation in Korea: A Multi-Institutional Comparison Study

**DOI:** 10.1155/2016/8576357

**Published:** 2016-07-21

**Authors:** Jinhong Jung, Moonkyoo Kong, Su Ssan Kim, Won Sup Yoon

**Affiliations:** ^1^Department of Radiation Oncology, Kyung Hee University Medical Center, Kyung Hee University School of Medicine, Seoul 02447, Republic of Korea; ^2^Department of Radiation Oncology, Asan Medical Center, University of Ulsan College of Medicine, Seoul 05505, Republic of Korea; ^3^Department of Radiation Oncology, Ansan Hospital, Korea University Medical Center, Ansan 15355, Republic of Korea

## Abstract

*Introduction*. To evaluate the dose distribution and coverage of axilla using only tangential field for whole breast radiotherapy (RT) at three institutions in Korea.* Methods*. We used computed tomography (CT) images of nine consecutive 1-2 sentinel lymph node-positive patients who underwent breast conserving surgery and whole breast RT without axillary lymph node (ALN) dissection for clinical T1-2N0 breast cancer. The CT data were transferred to three radiation oncologists in 3 institutions and each radiation oncologist created treatment plans for all nine patients; a total of 27 treatment plans were analyzed.* Results*. The mean doses delivered to levels I and II were 31.9 Gy (9.9–47.9 Gy) and 22.3 Gy (3.4–47.7 Gy). Ninety-five percent of levels I and II received a mean dose of 11.8 Gy (0.4–43.0 Gy) and 3.0 Gy (0.3–40.0 Gy). The percent volumes of levels I and II covered by 95% of the prescribed dose were only 29.0% (0.2–74.1%) and 11.5% (0.0–70.1%). The dose distribution and coverage of axilla were significantly different between three institutions (*p* = 0.001).* Conclusion*. There were discrepancies in ALN coverage between three institutions. A standardization of whole breast RT technique through further research with a nationwide scale is needed.

## 1. Introduction

The American College of Surgeons Oncology Group (ACOSOG) Z0011 trial showed that patients with early breast cancer and limited sentinel node involvement receiving lumpectomy, whole breast radiotherapy (RT), and adjuvant systemic treatment no longer require axillary lymph node dissection (ALND) [1, 2]. Considering that the regional recurrence rate after sentinel lymph node dissection (SLND) alone was <1%, despite the fact that 27.3% of patients had additional disease in the undissected axillary nodes, not all axillary occult diseases manifest as failure. However, there was a nontrivial percentage of patients treated contrary to protocol requirements; more than half of patients received RT coverage on not only the whole breast but also axillary lymph node (ALN) [3]. Therefore, a value of ALN coverage by tangential field for whole breast RT was still one of the most curious questions to physicians who treat a breast cancer.

Previous studies have explored ALN coverage of the standard or high tangential field for whole breast RT [4–13], and the axillary mean doses were 20–50 Gy for axilla level I and 3–47 Gy for level II [4–8]. Dose depends on the somatotype, such as convexity of chest wall, axillary vein route, and fatness, as well as RT technique, such as standard tangential field, high tangential field, and/or field-in-field using multileaf collimator modification, according to physician preference [4, 9, 11]. However, discrepancies in ALN coverage in same patient between institutions have not yet been explored. Furthermore, there was only one study reporting axilla coverage in Korean women [14].

In this study, we evaluated the dose distribution and coverage of axilla levels I-II using only the tangential field for whole breast RT with same patient simulation data at three institutions (Kyung Hee University Medical Center, Asan Medical Center, and Korea University Medical Center) in Korea.

## 2. Materials and Methods

### 2.1. Patients

We used computed tomography (CT) images of nine consecutive one to two SLN-positive breast cancer patients who underwent breast conserving surgery (BCS) and whole breast RT without ALND for their clinical T1-T2N0 breast cancer at the Kyung Hee University Medical Center. All patients had a tumor ≤50 mm in greatest dimension with a median tumor diameter of 2.2 cm. Four patients had a right-sided and 5 had a left-sided tumor.

### 2.2. Study Design

Patients were placed supine with both arms up and both hands holding on to a support during CT simulation. CT scan images with 5 mm sections were obtained and the CT data were transferred to each of the three radiation oncologists at 3 different institutions (Kyung Hee University Medical Center, Asan Medical Center, and Korea University Medical Center). The three radiation oncologists were randomly selected among radiation oncologists with more than 10 years of experience treating breast cancer patients at 3 institutions. Each radiation oncologist made treatment plans for all nine patients using tangential field to whole breast. Tangential fields were determined according to physician preference, and the superior borders of these fields intended to treat the breast only, without regard to nodal coverage. A dose of 50 Gy was delivered in 25 fractions to the whole breast. Finally, a total of 27 plans were analyzed. One of the three radiation oncologists contoured all axillary nodal volumes and organs at risk using the Danish guidelines [15].

### 2.3. Statistical Analyses

Mean dose, dose delivered to 95% and 50% of the axillary levels (*D*_95%_ and *D*_50%_), percent volume of the axillary levels receiving 95% and 50% of the prescribed dose (*V*_*D*95%_ and *V*_*D*50%_), and percent volume of the ipsilateral lung receiving 20 Gy, 10 Gy, and 5 Gy (*V*_20 Gy_, *V*_10 Gy_, and *V*_5 Gy_) were calculated using dose-volume histograms. Percent volume of the heart receiving 30 Gy (*V*_30 Gy_) and mean dose received by heart were calculated for the 5 patients with left-sided breast cancer. These values were compared between the three institutions by one-way analyses of variance with Tukey's post hoc tests. Associations between the distance from the humeral head to the superior border of the tangential field (DHS) with dose-volume parameters were analyzed by univariate linear regression. A *p* value <0.05 was considered to be statistically significant. All statistical analyses were performed using the SPSS statistical package (version 18.0, SPSS Inc., Chicago, IL).

## 3. Results

### 3.1. Level I and II Coverage

All plans had adequate coverage to the breast, defined as 95% of the breast volume receiving at least 95% of the prescribed dose. The mean volumes of axillary levels I and II were 56.0 cm^3^ (range 24.0–96.3 cm^3^) and 27.7 cm^3^ (range 12.5–45.0 cm^3^), respectively. The mean doses delivered to levels I and II were 31.9 Gy (range 9.9–47.9 Gy) and 22.3 Gy (3.4–47.7 Gy), respectively. Ninety-five percent of levels I and II received a mean dose of 11.8 Gy (range 0.4–43.0 Gy) and 3.0 Gy (0.3–40.0 Gy), respectively, and the percent volumes of levels I and II covered by the 95% of prescribed dose were only 29.0% (range 0.2–74.1%) and 11.5% (0.0–70.1%), respectively. The dose and volume results are summarized in Table 1. The mean DHS was 2.4 cm. In 3 of the 27 treatment plans, the superior border of the tangential field was set above the humeral head. Doses delivered to levels I and II and volumes received by radiation were related to DHS. When the superior border of the tangential field was closer to the humeral head, the axillary coverage increased (level I mean dose, *p* < 0.001; level I *D*_95%_, *p* < 0.001; level I *D*_50%_, *p* < 0.001; level I *V*_*D*95%_, *p* < 0.001; level I *V*_*D*50%_, *p* < 0.001; level II mean dose, *p* < 0.001; level II *D*_95%_, *p* = 0.024; level II *D*_50%_, *p* < 0.001; level II *V*_*D*95%_, *p* = 0.011; level II *V*_*D*50%_, *p* < 0.001). There was no significant difference in ALN coverage between right- and left-sided tumors.

### 3.2. Comparison between the Three Institutions

In all parameters related to levels I and II, except *D*_95%_ to level II, there were significant discrepancies between the three institutions (Table 2). The ALN coverage was lower in institutions A and B than in institution C. The mean dose delivered to ALN in institution C was significantly higher than in institutions A and B (level I, 44.8 Gy versus 23.8 Gy versus 27.0 Gy; level II, 35.4 Gy versus 12.9 Gy versus 18.6 Gy).

DHS was also significantly different between the institutions (*p* = 0.001). The mean DHS in institutions A and B was 3.6 cm and 2.9 cm, respectively, but was 0.7 cm in institution C. There were two cases in institution C where the superior border of the tangential field was set above the humeral head. Institution C used a high tangential field, which was defined with the superior border of the tangential field set <2 cm below the humeral head in all plans. In contrast, institutions A and B used a high tangential field in two cases, respectively.

The mean doses and the percentage volumes of ipsilateral lung receiving 5 Gy (*V*_5 Gy_), 10 Gy (*V*_10 Gy_) were significantly higher in institution C than in institutions A and B. However, there was no significant statistical discrepancy between the three institutions for the mean heart dose and *V*_30 Gy_.

## 4. Discussion

The ACOSOG Z0011 trial concluded that SLND without ALND can offer excellent regional control and equivalent survival, so it may be reasonable management for selected patients with early-stage breast cancer treated with BCS and adjuvant systemic therapy [1, 2]. The trial also demonstrated that adverse surgical effects were reported in 70% of patients after SLND + ALND and in 25% after SLND alone [16]. The use of SLND + ALND resulted in more wound infections, axillary seromas, and paresthesias than SLND alone. The EORTC AMAROS trial, which enrolled patients with T1-2 primary breast cancer and no palpable lymphadenopathy (744 ALND group and 781 axillary RT group), was another important study showing equivalent regional control and survival of axillary RT without ALND [17]. However, there was a significant difference in the incidence and severity of lymphedema in favor of the axillary RT group (5-year incidence of clinical signs of lymphedema in the ipsilateral arm, 23% versus 11%, *p* < 0.0001). The practice pattern was changed after these studies which showed excellent axillary control and lower treatment related complications [18], and, in the treatment setting that omits ALND, the ALN coverage of RT needed further investigation.

In the present study, we evaluated the dose distribution and coverage of axillary levels I-II using the tangential field for whole breast RT at three institutions in Korea. The tangential field planned for breast RT does not allow for adequate coverage of the axilla. The mean doses delivered to levels I and II were 31.9 Gy (range 9.9–47.9 Gy) and 22.3 Gy (3.4–47.7 Gy), respectively. Only 29.0% of level I and 11.5% of level II were covered by the 95% prescription dose. Results of the present study correspond with earlier studies, which reported insufficient axillary coverage of the tangential field [4–8]. We also found discrepancy in axillary coverage between the three institutions when we compared data from same CT image and axillary contour. Institution C had greater axillary coverage than institutions A and B in all dose-volume parameters, except *D*_95%_ to level II. To the best of our knowledge, this is the first study that statistically evaluated discrepancies in axillary coverage with the same patient data between multiple institutions.

ALN coverage by the tangential field for whole breast RT had discrepancies according to a patient's anatomical features and physician preference. Previous studies reported a broad range of ALN coverage by the tangential field [4, 6, 14]. Belkacemi et al. [6] showed that mean doses ranged from 1 to 57 Gy (median 22 Gy) to level I and 0–46 Gy (median 4 Gy) to level II. In a study by Alço et al. [4], the ranges of mean doses delivered to levels I and II were 16.7–50.4 Gy and 4.5–50.27 Gy, respectively. Because each whole breast RT was planned with consideration of patient's chest wall convexity and breast contour rather than the axillary vein route, these results, which showed a broad range of axillary coverage by the tangential field, were natural. Furthermore, DHS was one of the most important factors affecting the discrepancy of axillary coverage. Reznik et al. [11] demonstrated that when high tangents are applied, the coverage improved significantly. In the present study, although we evaluated axillary coverage using the same CT image for planning in each institution, DHS was significantly different between the institutions. Institution C had higher superior borders of the tangential field than did institutions A and B. These results suggested that the tangential field for breast cancer, especially the superior border of the field, could vary significantly between physicians. These differences in RT field also appeared in the Z0011 trial, which was conducted with a well-designed protocol. The study of RT field design in the Z0011 trial demonstrated that, of the 228 patients with detailed RT records, 18.9% received directed regional nodal RT using at least three fields and 51% received high tangential field RT [3].

Advanced RT technique such as high tangential field and/or field-in-field using multileaf collimator modification improved ALN coverage [4, 9, 11]. From the adequate ALN coverage view, the use of direct fields like in the AMAROS trial could be considered. However, whether such treatment might benefit patients with minimal axillary disease detected on SLND remains unknown, as we currently lack the data to draw conclusions about the relationship between ALN coverage and axillary tumor control. Indeed, not all ALN metastases develop into clinically detectable disease [1]. In the Z0011 trial, based on the finding that 27% of patients in the ALND arm had additional nodal metastases, patients randomized to the SLND alone arm were likely to have residual non-SN metastasis that was not removed by operation. Finally, the regional recurrence rates were similar between the 2 groups. Therefore, further study to define the optimal field and to reveal the relationship between ALN coverage and axillary tumor control should be conducted. The first step of this might be the standardization of the RT field.

The present study had several limitations. We analyzed nine patients with 27 treatment plans. Thus, it did not represent a standard somatotype of the Korean women. Another limitation is that our study was performed using only three radiation oncologists with experience in the treatment of breast cancer in 3 institutions. Despite these limitations, the present results provide valuable information about ALN coverage of the tangential field and the discrepancies between institutions, which could serve to motivate further research on standard RT field with a nationwide scale.

## 5. Conclusion

In conclusion, the tangential field for whole breast RT showed insufficient ALN coverage. There was a discrepancy of ALN coverage between the three institutions. A standardization of RT field through further research with a nationwide scale is needed.

## Figures and Tables

**Table 1:** Coverage of axillary levels I and II for a total of 27 plans.

Targets	Dose-volume parameters	Mean (range)
DHS	Mean length (cm)	2.4 (−1.0^*∗*^–6.0)

Level I	Mean dose (Gy)	31.9 (9.9–47.9)
	*D*_95%_ (Gy)	11.8 (0.4–43.0)
	*D*_50%_ (Gy)	30.9 (1.1–48.8)
	*V*_*D*95%_ (%)	29.0 (0.2–74.1)
	*V*_*D*50%_ (%)	64.5 (14.9–100)

Level II	Mean dose (Gy)	22.3 (3.4–47.7)
	*D*_95%_ (Gy)	3.0 (0.3–40.0)
	*D*_50%_ (Gy)	19.3 (0.7–48.6)
	*V*_*D*95%_ (%)	11.5 (0.0–70.1)
	*V*_*D*50%_ (%)	45.4 (5.1–100)

DHS: distance from the humeral head to the superior border of the tangential field; *D*_95%_: dose delivered to 95% of the target; *D*_50%_: dose delivered to 50% of the target; *V*_*D*95%_: percent volume of the target receiving 95% of the prescribed dose; *V*_*D*50%_: percent volume of the target receiving 50% of the prescribed dose.

^*∗*^Minus value means that the superior border of the tangential field was set above the humeral head.

**Table 2:** Dose-volume parameters comparison between the three institutions.

Targets	Dose-volume parameters	Mean (range)			*p*
		Institution A	Institution B	Institution C	
DHS	Mean length (cm)	3.6 (1.0–6.0)	2.9 (0.5–4.5)	0.7 (−1.0–2.0)^*∗*^	0.001

Level I	Mean dose (Gy)	23.8 (9.9–37.8)	27.0 (16.1–42.1)	44.8 (39.8–47.9)	<0.001
	*D*_95%_ (Gy)	2.6 (0.4–10.1)	3.3 (1.0–12.2)	29.6 (5.7–43.0)	0.001
	*D*_50%_ (Gy)	19.9 (1.1–44.5)	25.4 (3.6–48.8)	47.4 (45.4–48.8)	0.001
	*V*_*D*95%_ (%)	13.0 (0.2–23.5)	26.4 (2.6–63.5)	47.5 (1.6–74.1)	0.002
	*V*_*D*50%_ (%)	47.0 (14.9–80.0)	52.0 (26.5–89.7)	94.6 (81.1–100)	<0.001

Level II	Mean dose (Gy)	12.9 (3.4–20.4)	18.6 (13.7–30.3)	35.4 (27.2–47.7)	<0.001
	*D*_95%_ (Gy)	1.0 (0.3–2.0)	1.1 (0.6–1.8)	7.0 (1.5–40.0)	0.324
	*D*_50%_ (Gy)	4.2 (0.7–16.3)	9.7 (1.8–43.3)	44.0 (36.7–48.6)	<0.001
	*V*_*D*95%_ (%)	1.6 (0.0–7.3)	11.4 (0.0–28.7)	21.5 (0.0–70.1)	0.014
	*V*_*D*50%_ (%)	25.0 (5.1–42.1)	36.4 (24.7–61.7)	74.9 (55.8–100)	<0.001

Lung	Mean dose (Gy)	6.3 (3.9–9.7)	6.7 (5.0–8.5)	8.3 (6.4–10.0)	0.021
	*V*_5 Gy_ (%)	17.4 (10.9–28.2)	21.9 (16.3–26.9)	28.7 (23.6–32.3)	<0.001
	*V*_10 Gy_ (%)	13.5 (8.3–20.9)	14.9 (10.5–19.5)	19.1 (14.2–23.3)	0.006
	*V*_20 Gy_ (%)	11.0 (6.3–16.8)	11.4 (7.6–15.5)	14.4 (9.8–18.9)	0.061

Heart^†^	Mean dose (Gy)	2.2 (0.9–3.3)	2.3 (1.2–3.5)	3.7 (1.1–7.8)	0.286
	*V*_30 Gy_ (%)	0.9 (0.0–2.0)	1.8 (0.3–3.7)	1.8 (0.0–3.3)	0.449

DHS: distance from the humeral head to the superior border of the tangential field; *D*_95%_: dose delivered to 95% of the target; *D*_50%_: dose delivered to 50% of the target; *V*_*D*95%_: percent volume of the target receiving 95% of the prescribed dose; *V*_*D*50%_: percent volume of the target receiving 50% of the prescribed dose; *V*_5 Gy_: percent volume of the lung receiving 5 Gy; *V*_10 Gy_: percent volume of the lung receiving 10 Gy; *V*_20 Gy_: percent volume of the lung receiving 20 Gy; *V*_30 Gy_: percent volume of the heart receiving 30 Gy.

^*∗*^Minus value means that the superior border of the tangential field was set above the humeral head.

^†^Percent volume of the heart receiving 30 Gy (*V*_30 Gy_) and mean dose received by heart were calculated for 5 patients with left-sided breast cancer.

## References

[1] Giuliano A. E., McCall L., Beitsch P. (2010). Locoregional recurrence after sentinel lymph node dissection with or without axillary dissection in patients with sentinel lymph node metastases: the American college of surgeons oncology group Z0011 randomized trial. *Annals of Surgery*.

[2] Giuliano A. E., Hunt K. K., Ballman K. V. (2011). Axillary dissection vs no axillary dissection in women with invasive breast cancer and sentinel node metastasis: a randomized clinical trial. *The Journal of the American Medical Association*.

[3] Jagsi R., Chadha M., Moni J. (2014). Radiation field design in the ACOSOG Z0011 (Alliance) trial. *Journal of Clinical Oncology*.

[4] Alço G., Iğdem S. I., Ercan T. (2010). Coverage of axillary lymph nodes with high tangential fields in breast radiotherapy. *The British Journal of Radiology*.

[5] Aristei C., Chionne F., Marsella A. R. (2001). Evaluation of Level I and II axillary nodes included in the standard breast tangential fields and calculation of the administered dose: results of a prospective study. *International Journal of Radiation Oncology, Biology, Physics*.

[6] Belkacemi Y., Allab-Pan Q., Bigorie V. (2013). The standard tangential fields used for breast irradiation do not allow optimal coverage and dose distribution in axillary levels I-II and the sentinel node area. *Annals of Oncology*.

[7] Belkacemi Y., Bigorie V., Pan Q. (2014). Breast Radiotherapy (RT) Using Tangential Fields (TgF): a prospective evaluation of the dose distribution in the Sentinel Lymph Node (SLN) area as determined intraoperatively by clip placement. *Annals of Surgical Oncology*.

[8] Krasin M., McCall A., King S., Olson M., Emami B. (2000). Evaluation of a standard breast tangent technique: a dose-volume analysis of tangential irradiation using three-dimensional tools. *International Journal of Radiation Oncology, Biology, Physics*.

[9] Ohashi T., Takeda A., Shigematsu N. (2009). Dose distribution analysis of axillary lymph nodes for three-dimensional conformal radiotherapy with a field-in-field technique for breast cancer. *International Journal of Radiation Oncology, Biology, Physics*.

[10] Reed D. R., Lindsley S. K., Mann G. N. (2005). Axillary lymph node dose with tangential breast irradiation. *International Journal of Radiation Oncology, Biology, Physics*.

[11] Reznik J., Cicchetti M. G., Degaspe B., Fitzgerald T. J. (2005). Analysis of axillary coverage during tangential radiation therapy to the breast. *International Journal of Radiation Oncology, Biology, Physics*.

[12] Takeda A., Shigematsu N., Ikeda T. (2004). Evaluation of novel modified tangential irradiation technique for breast cancer patients using dose-volume histograms. *International Journal of Radiation Oncology, Biology, Physics*.

[13] Takeda A., Shigematsu N., Kondo M. (2000). The modified tangential irradiation technique for breast cancer: how to cover the entire axillary region. *International Journal of Radiation Oncology, Biology, Physics*.

[14] Park S.-H., Kim J.-C., Lee J. E., Park I.-K. (2015). Virtual lymph node analysis to evaluate axillary lymph node coverage provided by tangential breast irradiation. *Radiation Oncology Journal*.

[15] Nielsen M. H., Berg M., Pedersen A. N. (2013). Delineation of target volumes and organs at risk in adjuvant radiotherapy of early breast cancer: national guidelines and contouring atlas by the Danish Breast Cancer Cooperative Group. *Acta Oncologica*.

[16] Lucci A., McCall L. M., Beitsch P. D. (2007). Surgical complications associated with sentinel lymph node dissection (SLND) plus axillary lymph node dissection compared with SLND alone in the American College of Surgeons Oncology Group trial Z0011. *Journal of Clinical Oncology*.

[17] Donker M., van Tienhoven G., Straver M. E. (2014). Radiotherapy or surgery of the axilla after a positive sentinel node in breast cancer (EORTC 10981-22023 AMAROS): a randomised, multicentre, open-label, phase 3 non-inferiority trial. *The Lancet Oncology*.

[18] Caudle A. S., Hunt K. K., Tucker S. L. (2012). American College of Surgeons Oncology Group (ACOSOG) Z0011: impact on surgeon practice patterns. *Annals of Surgical Oncology*.

